# Modelling the skeletal muscle injury recovery using *in vivo* contrast-enhanced micro-CT: a proof-of-concept study in a rat model

**DOI:** 10.1186/s41747-020-00163-4

**Published:** 2020-06-03

**Authors:** Bruno Paun, Daniel García Leon, Alex Claveria Cabello, Roso Mares Pages, Elena de la Calle Vargas, Paola Contreras Muñoz, Vanessa Venegas Garcia, Joan Castell-Conesa, Mario Marotta Baleriola, Jose Raul Herance Camacho

**Affiliations:** 1 Medical Molecular Imaging Group, Vall d’Hebron Research Institute (VHIR), CIBER-BBN, CIBBIM-Nanomedicine, ISCIII, Hospital Universitari Vall d’Hebron, Universitat Autònoma de Barcelona (UAB), Passeig de la Vall d’Hebron 119-129, 08035 Barcelona, Spain; 2 Health & Biomedicine division, Leitat Technological Center, 2. C/ Pallars, 179-185, 08005 Barcelona, Spain; 3 Bioengineering, Cell therapy and Surgery in Congenital Malformations Laboratory, Vall d’Hebron Research Institute (VHIR), Hospital Universitari Vall d’Hebron, Universitat Autònoma de Barcelona (UAB), Passeig de la Vall d’Hebron 119-129, 08035 Barcelona, Spain

**Keywords:** Muscle (skeletal), Muscular diseases, Rats, Tomography (x-ray computed), Wound healing

## Abstract

**Background:**

Skeletal muscle injury characterisation during healing supports trauma prognosis. Given the potential interest of computed tomography (CT) in muscle diseases and lack of *in vivo* CT methodology to image skeletal muscle wound healing, we tracked skeletal muscle injury recovery using *in vivo* micro-CT in a rat model to obtain a predictive model.

**Methods:**

Skeletal muscle injury was performed in 23 rats. Twenty animals were sorted into five groups to image lesion recovery at 2, 4, 7, 10, or 14 days after injury using contrast-enhanced micro-CT. Injury volumes were quantified using a semiautomatic image processing, and these values were used to build a prediction model. The remaining 3 rats were imaged at all monitoring time points as validation. Predictions were compared with Bland-Altman analysis.

**Results:**

Optimal contrast agent dose was found to be 20 mL/kg injected at 400 μL/min. Injury volumes showed a decreasing tendency from day 0 (32.3 ± 12.0mm^3^, mean ± standard deviation) to day 2, 4, 7, 10, and 14 after injury (19.6 ± 12.6, 11.0 ± 6.7, 8.2 ± 7.7, 5.7 ± 3.9, and 4.5 ± 4.8 mm^3^, respectively). Groups with single monitoring time point did not yield significant differences with the validation group lesions. Further exponential model training with single follow-up data (*R*^2^ = 0.968) to predict injury recovery in the validation cohort gave a predictions root mean squared error of 6.8 ± 5.4 mm^3^. Further prediction analysis yielded a bias of 2.327.

**Conclusion:**

Contrast-enhanced CT allowed *in vivo* tracking of skeletal muscle injury recovery in rat.

## Key points

- Contrast-enhanced micro-computed tomography showed applicability in monitoring skeletal muscle injury recovery.
- Highest accuracy in lesion recovery predictions was obtained on day 14 after injury.
- We provided a proof of concept to track skeletal muscle healing stages using CT-derived volume-based lesion recovery.

## Background

Skeletal muscle (SM) represents about 40% of the body mass and is formed by contractile multinucleated muscle fibres as a result from myoblasts fusion. Injuries in SM may come from diverse events, including direct trauma from muscle contusions, lacerations and strains or indirect trauma from degenerative diseases like muscular dystrophy, among others. Professional athletes are the most affected by SM injuries, representing from 10 to 55% of all injuries in this population [1, 2]. Moreover, the type, severity, size and location of the injury make inference of recovery and rehabilitation times challenging, thus directly affecting prognosis of lay-off times [3].

In mammals, SM has the ability to regenerate in response to injury [4]. This process can be divided into three phases [1, 4–6]: (1) *degeneration*, characterised by muscle fibres rupture and necrosis, formation of hematoma and inflammatory cell reaction; (2) *regeneration*, characterised by phagocytosis of the damaged tissue, satellite cells (SM stem cells) activation and angiogenesis; and (3) *remodelling*, characterised by the maturation and reorganisation of regenerated muscle fibres, recovery of muscle function together with fibrosis and scar tissue formation. The new vascular network emerges from surviving trunks of the blood vessels into the injured region providing means for oxygen delivery and nutrients necessary to recover mature myofibres [1, 7].

To characterise *in vivo* muscle injury, both magnetic resonance imaging (MRI) and ultrasound are well-established imaging approaches in hospitals [8]. However, there is a knowledge gap regarding the approaches to monitor and quantify SM injury using computed tomography (CT), which plays an important role in tissue density characterisation in muscle diseases [9]. Along with its application in whole-body scans, CT is also part of hybrid scanners as single-photon computed tomography/CT or positron emission tomography (PET)/CT where functional studies can be conducted to monitor SM lesion healing.

To the best of our knowledge, there are no studies where CT has been used for *in vivo* tracking of SM injury. There are several studies in the literature [7, 10], where the authors use *ex vivo* contrast-enhanced micro-CT to quantify underlying angiogenesis in the process of wound healing. Therefore, the lack of methodologies allowing *in vivo* assessment of SM recovery was the main motivation of the present work.

Consequently, we aimed to (i) present a new methodology to image and quantify *in vivo* SM recovery by contrast-enhanced micro-CT means and (ii) propose a proof-of-principle study of a SM recovery prediction model.

## Methods

### Animal preparation

The study was approved by the Local Animal Ethics Committee and was performed in accordance with Spanish (RD 53/2013) and European (2010/63/UE) legislation. Wistar male adult rats (*n* = 27), weighing approximately 425 g each (425.4 ± 43 g), were housed at 22–24 °C, maintained on a 12-h light/dark cycle, and used for the experiments. Water and food were given *ad libitum* during the experiments. Anaesthesia was induced with 4% isoflurane (Aerrane, Baxter Healthcare, Deerfield, IL, USA) and maintained with 1.5–2.0% isoflurane in O_2_ at 1 L/min during the whole study. A 22-gauge intravenous cannula (Introcan Safety® IV Catheter, 22G, B. Braun, Melsungen, Germany) was placed in the tail vein for iopamidol (iopamiro 370 mg/mL, Bracco Imaging, Milan, Italy) administration as contrast agent (CA) by means of an infusion pump (Harvard apparatus PHD 2000 Infusion, Holliston, MA, USA). Post-surgically, 250 mL of water with 10% of buprenorphine (buprecare 0.3 mg/mL, Divasa Farmavic, Barcelona, Spain) was given to all operated animals.

### Contrast agent dose optimisation

Optimal CA dose to distinguish between injury and neighbouring tissue was first determined by performing a contrast enhancement study in a separate set of experiments including five rats. These were placed in the micro-CT bed, and five different infusion rates (100, 200, 300, 400, and 500 μL/min) were tested for 20 min to obtain different contrast-to-noise ratios (CNR) in acquired images. Imaging parameters were as follows: field of view 40 mm, voxel size  80 μm (isotropic), kVp 90, tube current 200 μA and exposure time 120 s. Images were acquired approximately every 2.5 min, resulting in total of 9 images per rat. Contrast enhancement was assessed in the femoral artery and neighbouring tissue by delineating circular volumes of interest of 1 mm in diameter, obtaining their mean density and standard deviation values. From these, we calculated CNR using following formula [11]:

$$ CNR=\frac{\left|{\mu}_{\mathrm{A}}-{\mu}_{\mathrm{T}}\right|}{\sqrt{\sigma_{\mathrm{A}}^2+{\sigma}_{\mathrm{T}}^2}}, $$

where *μ*_A_ and *μ*_T_ represent mean density values of volumes of interest in femoral artery and tissue area, respectively, and *σ*_A_ and *σ*_T_ represent their standard deviations. Optimal infusion rate providing adequate image contrast was set following the Rose criterion (CNR > 5), as previously described [12].

After CA administration, the pump was stopped, and micro-CT images were acquired. One millilitre of saline solution was injected after image acquisition was finished.

### Skeletal muscle injury model

For the injury model, we used a recently developed surgically induced SM injury rat model proposed by Contreras-Muñoz et al [6]. Briefly, 23 anaesthetised rats were immobilised by the fixation of tail and extremities with adhesive strips to a styrofoam surface exposing the ventral side of the right crus. Skeletal traumatic muscle injuries were induced in the rat medial gastrocnemius muscle by a 18-gauge biopsy needle (Bard® Monopty® Disposable Core Biopsy Instrument, Bard Biopsy Systems, Tempe, USA) with a 0.84-mm inner diameter. Transversal biopsy procedure was performed at the muscle-tendon junction level of the left leg medial gastrocnemius muscle (3 mm from the start of muscle-tendon junction and 2 mm in depth). Immediately after muscle injury, a cannula (Introcan Safety® IV Catheter, 22G, B. Braun, Melsungen, Germany) was introduced within the injury in order to proceed with the micro-CT imaging protocol.

### Micro-CT

Micro-CT studies were performed using a Quantum FX micro-CT scanner (PerkinElmer, Hopkinton, MA, USA). Rats (*n* = 23) were positioned on their right side in a bed, and their left leg was immobilised to minimise possible involuntary movement and motion-related artefacts. During the scans, rats were kept under anaesthesia, and CA was administered for 20 min as described above. Two images were acquired at the same day when muscle injury was induced: first an image of the injury with the cannula at 14 min after the beginning of CA infusion for location purposes, and then, after removing the cannula from the injury, a second image was acquired at 20 min right after stopping the CA injection. Again, 1 mL of saline solution was injected after image acquisition was finished to ease contrast clearance.

To image lesion recovery at different time points, 20 rats were sorted into 5 different groups (*n* = 4 per group) according to the follow-up day at 2, 4, 7, 10, or 14 days after injury, respectively. Additional 3 rats were imaged at all mentioned follow-up days as validation. High-resolution images were acquired so that the centre of the field of view was aligned with the middle of the fibula in sagittal and coronal planes, thus covering the maximal region of the left limb containing the injury. Experimental imaging parameters were field of view 40 mm, voxel size 80 μm (isotropic), kVp 90, tube current 200 μA and exposure time 270 s. All images were reconstructed using a filtered back-projection approach with a Ram-Lak filter, including a ring reduction algorithm [13].

### Image processing

Greyscale values between image datasets were normalised by an histogram matching algorithm implemented in the Insight Toolkit [14]. All image datasets in the study were normalised by matching their histograms to the reference image histogram, which was chosen to be the injury image of rat 1 at day 0. Adapted normalised image datasets were then filtered in Fiji [15] using a non-local means denoising filter [16, 17]. Noise standard deviation was set to 2 with smoothing factor set to 1. Contrast-enhanced anatomy, together with high-intensity anatomy like bones, was segmented from denoised images using thresholding based on Renyi entropy [18] with min and max values set to 77 and 255, respectively. These values were experimentally derived from denoised images to ease the injury segmentation process. All segmented contrast-enhanced anatomy images were then manually processed in three-dimensional slicer (version 4.9.0) [19]. The injury was further segmented from contrast-enhanced anatomy mask, excluding surrounding adipose tissue and vasculature. Once the refined SM injury mask was obtained, injury volume was calculated as the mask voxel number multiplied by voxel volume.

### Lesion recovery prediction model

Single follow-up day cohort data (*n* = 20) were used to feed an exponential model of SM injury recovery. The generalised exponential model used was *Vt* = *V*_0_ × exp (*b* × *t*), where *t* is the corresponding post-injury time (days), *V*_0_ is the initial injury size (mm^3^) and *b* is the healing rate (days^−1^). Model was trained using the least absolute residual method available in Matlab (The Mathworks Inc., Natick, MA, USA), quantifying both *R*^2^ and root mean squared error. Afterwards, injury volumes were predicted in the validation cohort (*n* = 3) at all monitored post-injury time points.

### Statistical analysis

Data are expressed as mean and standard deviation. Injury volumes and model predictions were compared using paired *t* test and Bland-Altman analysis after testing for normality. All statistical analyses were performed using the Graphpad Prism software (Graphpad Software Inc., San Diego, CA, USA). A *p* value below 0.05 was considered as statistically significant.

## Results

### Optimal contrast agent dose

**Fig. 1:**
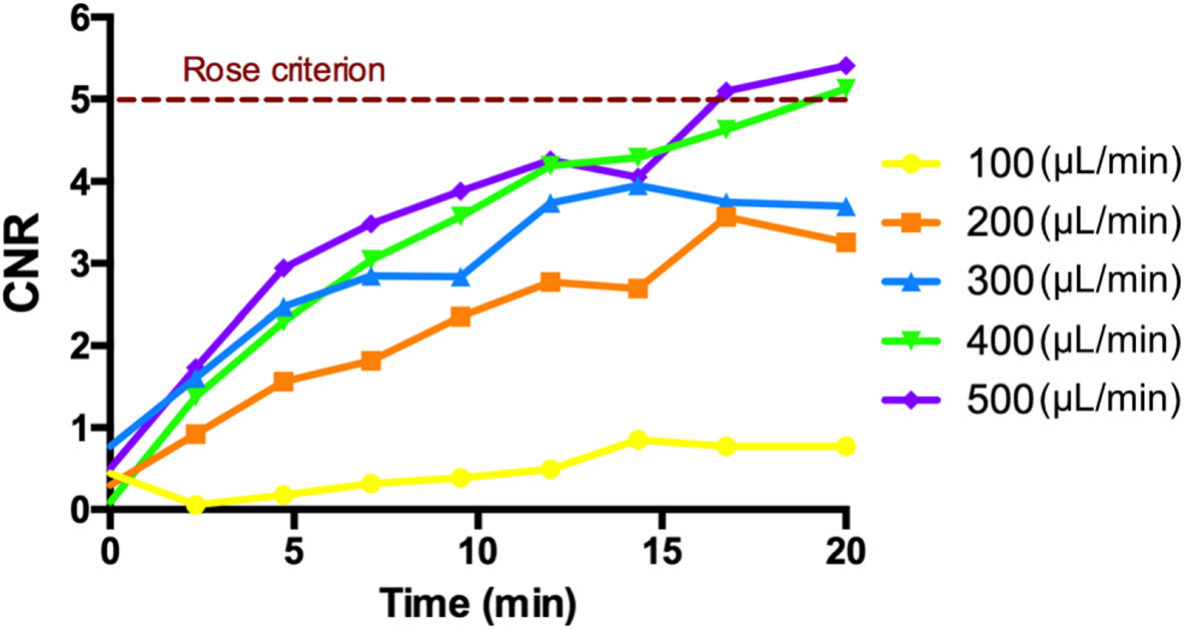
Contrast-to-noise ratio quantification for several infusion rates. Tested contrast injection rates from 100 to 500 μL/min (yellow), with an overall administration time of 20 min. The red dashed line defines the applied cutoff criterion to determine the optimal infusion rate. *CNR* Contrast-to-noise ratio

Prior to tracking injury healing by micro-CT, analysis of the influence of CA dose in image contrast was performed to determine optimal infusion rate. Figure 1 shows obtained CNR curves for each tested infusion rate. Since cutoff criterion was set at CNR over 5 after 20 min of infusion, contrast injection rates of 400 and 500 μL/min were the only ones satisfying this requirement. However, to reduce the amount of CA injected to animals and to avoid possible side effects, 400 μL/min was chosen as optimal rate and was used in following study, resulting in total administered amount of contrast of 20 mL/kg per rat.

### *In vivo* SM lesion tracking

**Fig. 2:**
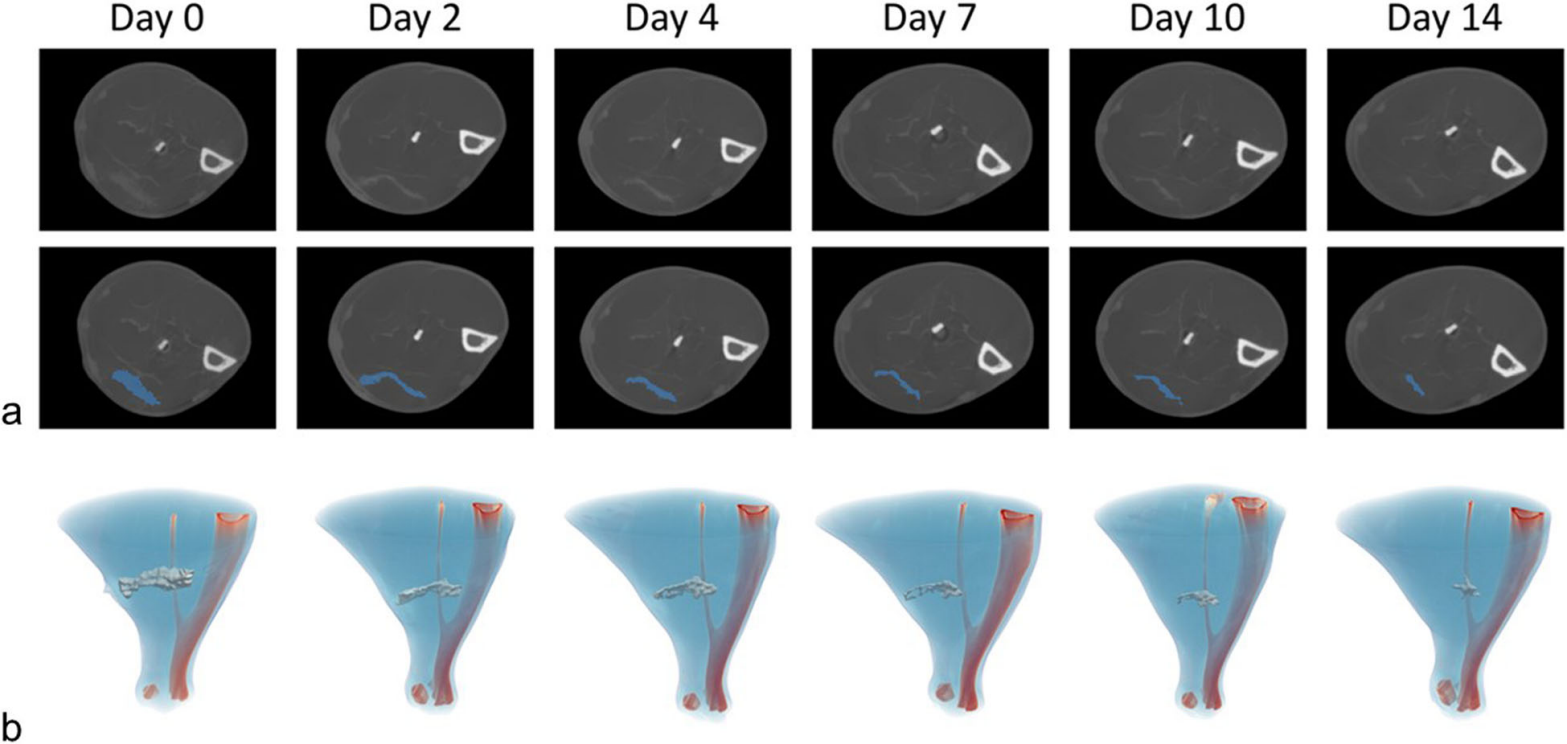
Contrast-enhanced micro-computed tomography images and segmented injuries from one representative example of the validation cohort. **a** First row shows short-axis images of the lesion in similar location during all follow-up time points. Images were reconstructed using a filtered back-projection approach with a Ram-Lak filter. Second row images superimpose the obtained segmentation mask (blue) of the injury on the first row images. **b** Three-dimensional volumetric rendering of the segmented lesions (white) during all follow-up time points

**Fig. 3:**
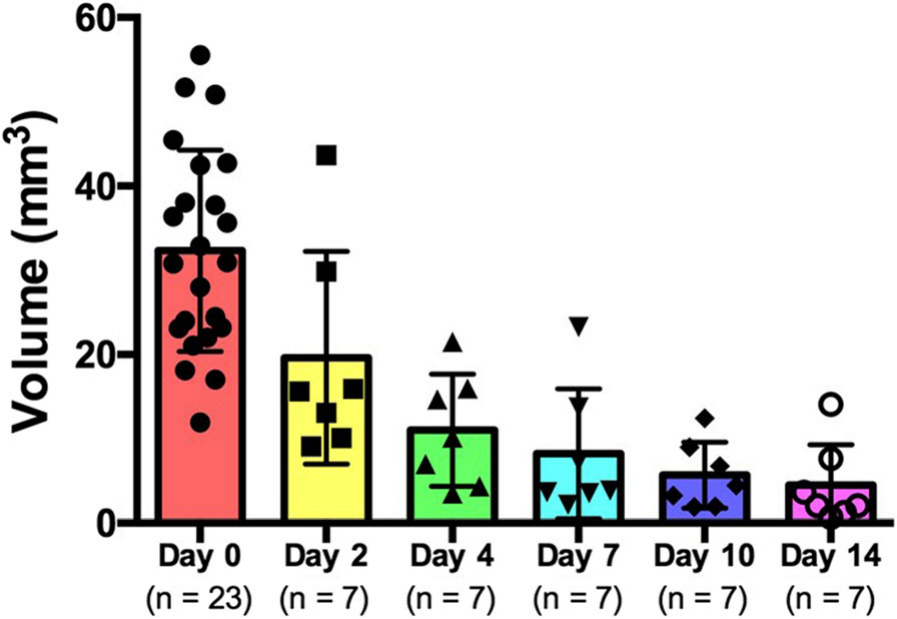
*In vivo* three-dimensional skeletal muscle injury volume quantification up to 14 days after injury. This quantification includes both single follow-up (*n* = 20) and validation (*n* = 3) cohorts

In Fig. 2, one example of SM lesion healing in short-axis view monitored at all post-injury time points is presented. As shown, lesion healing process remains rather active up to 2 weeks after injury. This was further quantified in Fig. 3, sorting lesion volumes according to the day injury was imaged. The mean initial lesion size (*n* = 23) was 32.3 ± 12.0 mm^3^. As expected, lesion healing process yielded decreasing injury volumes (*n* = 7) at day 2 (19.6 ± 12.6 mm^3^), day 4 (11.0 ± 6.7 mm^3^), day 7 (8.2 ± 7.7 mm^3^), day 10 (5.7 ± 3.9 mm^3^), and day 14 (4.47 ± 4.8 mm^3^) post-injury. Additionally, groups with a single monitored time point after injury (5 time points, *n* = 4 each) were compared to the validation cohort (*n* = 3) to support this decreasing tendency. Indeed, lesion volumes did not show any statistically significant differences between single time point groups and validation group at day 0 (34.0 ± 11.9 *versus* 21.4 ± 3.8 mm^3^, *p* = 0.088), day 2 (22.1 ± 14.5 *versus* 16.3 ± 11.7 mm^3^, *p* = 0.598), day 4 (11.3 ± 5.3 *versus* 10.7 ± 9.6 mm^3^, *p* = 0.915), day 7 (9.2 ± 9.7 *versus* 7.0 ± 5.9 mm^3^, *p* = 0.744), day 10 (6.4 ± 4.5 *versus* 4.7 ± 3.8 mm^3^, *p* = 0.622) and at day 14 (3.5 ± 3.0 *versus* 5.8 ± 0.4 mm^3^, *p* = 0.584), respectively.

### Injury volume predictions

**Fig. 4:**
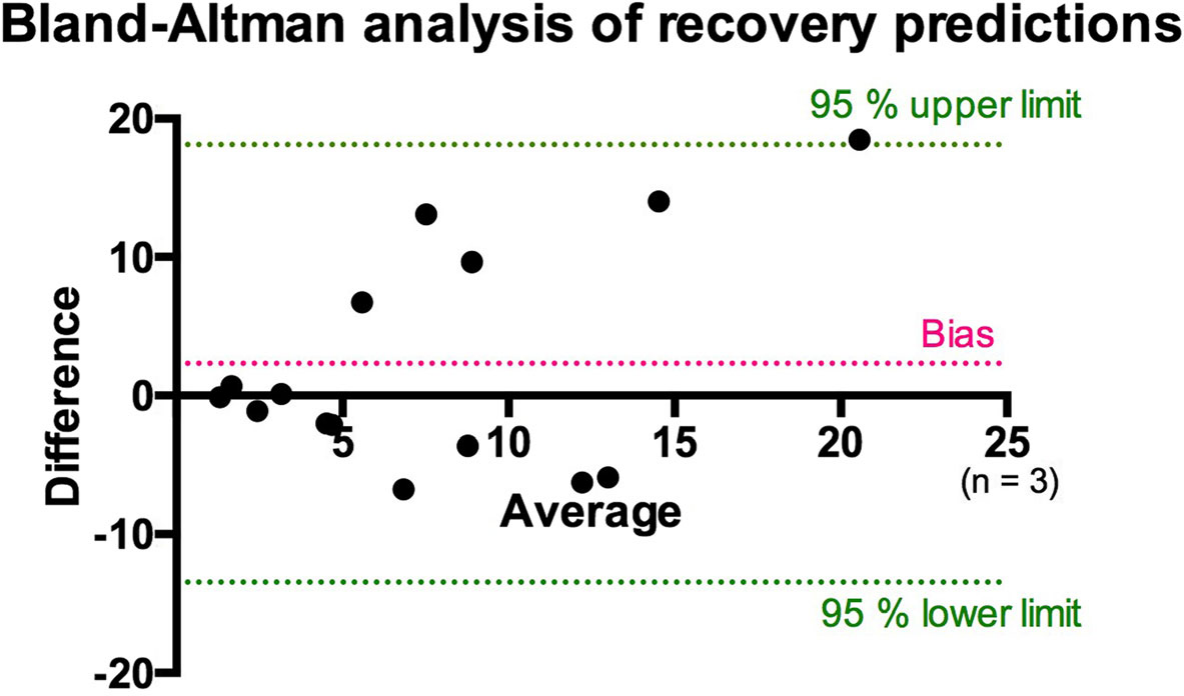
Bland-Altman analysis of the predicted injury volumes in the validation cohort. Predictions were compared to the injury volumes during all follow-up time points. Red dotted line corresponds to the bias, with corresponding lower and upper limits of agreement at 95% represented with green dotted lines

Model training with single follow-up data yielded a correlation of *R*^2^ = 0.968, with root-mean-square error = 1.77 mm^3^. This was further tested with the validation cohort (*n* = 3) at all monitored time points (days 2, 4, 7, 10, and 14 after injury). Mean root-mean-square error in predictions was 6.8 ± 5.4 mm^3^. As presented in the Bland-Altman plot (Fig. 4), overall predictions yielded a bias of 2.3 (limits of agreement at 95%, -13.5 and 18.1). The exponential model better performed at low average values, offering higher accuracy for nearly healed injury time points.

## Discussion

In this proof-of-concept study, we assessed the *in vivo* CT applicability to track the progression of SM injury recovery. For this purpose, we previously defined a contrast enhancement CT protocol in order to guarantee proper differentiation between injury and healthy tissue. After quantifying injury volumes during follow-up to track injury recovery, an exponential prediction model of injury recovery has been proposed to mimic the observed decreasing tendency during healing. Although simple, the model offered a higher accuracy for late follow-up time points than that for early time points when the lesion is nearly recovered.

Regarding the contrast enhancement protocol, we found a CA volume of 20 mL/kg that was necessary to obtain a proper CNR on CT images. Injecting more contrast may cause unnecessary side effects in rats without significant increasing image contrast. In addition, the proposed injection rate of 400 μL/min was found to be the best rate allowing a proper biodistribution of the CA without causing any side effect in rats. This experimentally determined CA amount was in agreement with guidelines about intravenous administration volumes for rats reported by Diehl et al [20].

The murine model of skeletal muscle injury used in this study has been previously described in detail [6]. As previously reported, this injury model well reproduces the human muscle strain injuries observed in professional athletes. This was characterised by means of histology and MRI (standard of care in muscle lesion tracking), showing the healing capacity observed in animal models of spontaneous skeletal muscle injury as the dystrophic mdx-mice [21]. Although similar looking skeletal muscle injuries on MRI at the same anatomical site, different healing rates have been observed as the lesion nature varies (either high-speed running or over-stretching) in athletes [22].

This study focused on the applicability of *in vivo* CT imaging to track SM lesion recovery. However, established imaging techniques for prognosis and muscle repair monitoring in the clinics are both MRI and ultrasound [8]. The latter is normally considered to be first choice for clinical diagnosis due to its low price and fast applicability, but MRI is preferred when detailed characterisation of injury is required [23]. Recently, Todeschini et al. [24] reported a series of 39 professional soccer players to study pubalgia imaging features with both ultrasound and MRI. Interestingly, from 9 individuals with a positive MRI scan regarding lesions of the common aponeurosis of the rectus abdominis/adductor longus muscles, only 4 yielded a positive ultrasound scan. MRI has been positioned as a widely used technique in muscle trauma evaluation, playing an increasingly role in grading injury severity and guiding return to play in the injured athlete [25]. Nonetheless, this technique does not offer any information regarding tissue dynamics when healing as a counterpart. Leaning on hybrid approaches using PET /CT or PET/MRI scans, this may be of relevance to study SM lesion healing dynamics in athletes.

There are just a few studies using PET/CT to assess muscle injury. For instance, Carter et al [26] studied metabolic changes related to thermally induced muscle injury in rabbits using 2-deoxy-2-[fluorine-18]fluoro-D-glucose (^18^F-fluorodeoxyglucose, ^18^F-FDG) PET. Xie et al [27] showed that mice with muscle injury induced by electroporation show increased uptake of ^64^Cu, thus demonstrating ^64^Cu-Cl_2_ PET/CT as a new molecular imaging technique for skeletal muscle. Recently, Pervaiz et al [28] showed that ^18^F-FDG PET/CT can be used for imaging inflammation and muscle injury caused by cholesterol crystal emboli. However, these studies focused on the use of radiopharmaceuticals to assess a specific physiological disorder that cannot explain the whole SM recovery. Merging these findings with *in vivo* contrast-enhanced CT quantifying muscle recovery, both imaging techniques may offer enriched lesion healing structural and functional information, yielding a more accurate injury prognosis, among other uses.

Although simple, this study also presented an exponential model of muscle recovery as a proof of concept. This model relied on quantified injury volumes through time to mimic the observed decreasing tendency derived from two-dimensional histological analyses in previous studies [6]. Yielded predictions were accurate when lesion volumes were nearly recovered but differed more at few days after injury. One way to better predict lesion recovery at all monitored time points could be to detect additional lesion features via convolutional neural network (CNN) models. These deep learning models can automatically learn hierarchical features from raw images to classify these according to a training classification [29]. For instance, Zhang and colleagues [30] presented a CNN model to classify soft tissue sarcoma grading using both CT and MR images from a series of 51 patients, offering great performance in this task. In this matter, CNNs could definitely be applied to track skeletal muscle injury healing, supporting current trauma prognosis.

This study has several limitations. First, there is a limited number of rats included in the validation cohort. Although tracking full progression of the injury recovery in all animals would have been desirable, no statistical differences were observed between the single follow-up day and the validation cohorts. Second, no histological analysis of the lesions was provided. Conversely, reported three-dimensional SM lesion characterisation data are consistent with previously reported histological results. Regarding lesion recovery, a direct association to professional athlete lesions cannot be established as the lesion nature varies from the surgically induced one in the rat model. Also, *in vivo* SM lesion tracking cannot be directly extrapolated to human studies. Indeed, the amount of iodine-based contrast agent used in our experiments exceeds the maximum recommended dose for contrast enhancement studies included in current clinical guidelines. To overcome the low soft tissue contrast problem during SM injury monitoring, early injury stages may offer a good starting point for tracking. For instance, both oedema and inflammatory processes in clinical contrast-enhanced CT images could serve as initial landmarks to ease injury segmentation process. Adapting this prior segmentation to subsequent post-injury examinations leaning on anatomical references detection, recovery predictions and the expert knowledge to refine lesion border delineation accordingly may offer similar outcomes as the ones we presented from animal experiments. Of note, acquired *z*-axis resolution in conventional CT scanners (~ 3 mm) might hinder this task, so detailed studies would be required to minimise the use of CA. Altogether, additional *in vivo* lesion tracking using contrast-enhanced CT in large animal models first, and then in patient studies, would be required to further validate these results.

In conclusion, our study showed that contrast-enhanced CT can allow *in vivo* tracking of SM healing after injury, presenting a proof of concept of an exponential recovery model, in a rat model.

## Data Availability

The datasets used and/or analysed during the current study are available from the corresponding author on reasonable request.

## Abbreviations

CA: Contrast agent
CNN: Convolutional neural network
CNR: Contrast-to-noise ratio
CT: Computed tomography
FDG: Fluorodeoxyglucose
MRI: Magnetic resonance imaging
PET: Positron emission tomography
SM: Skeletal muscle
