# Correction to: Modelling the skeletal muscle injury recovery using *in vivo* contrast-enhanced micro-CT: a proof-of-concept study in a rat model

**DOI:** 10.1186/s41747-020-00202-0

**Published:** 2020-12-17

**Authors:** Bruno Paun, Daniel García Leon, Alex Claveria Cabello, Roso Mares Pages, Elena de la Calle Vargas, Paola Contreras Muñoz, Vanessa Venegas Garcia, Joan Castell-Conesa, Mario Marotta Baleriola, Jose Raul Herance Camacho

**Affiliations:** 1 Medical Molecular Imaging Group, Vall d’Hebron Research Institute (VHIR), CIBER-BBN, CIBBIM-Nanomedicine, ISCIII, Hospital Universitari Vall d’Hebron, Universitat Autònoma de Barcelona (UAB), Passeig de la Vall d’Hebron 119-129, 08035 Barcelona, Spain; 2 Health & Biomedicine division, Leitat Technological Center, 2. C/ Pallars, 179-185, 08005 Barcelona, Spain; 3 Bioengineering, Cell therapy and Surgery in Congenital Malformations Laboratory, Vall d’Hebron Research Institute (VHIR), Hospital Universitari Vall d’Hebron, Universitat Autònoma de Barcelona (UAB), Passeig de la Vall d’Hebron 119-129, 08035 Barcelona, Spain; 4 Medical Molecular Imaging Group, Vall d’Hebron Research Institute (VHIR), CIBER-BBN, CIBBIM-Nanomedicine, ISCIII, Hospital Universitari Vall d’Hebron, Universitat Autònoma de Barcelona (UAB), Passeig de la Vall d’Hebron 119-129, 08035 Barcelona, Spain

**Correction to: Eur Radiol Exp 4, 33 (2020)**

**https://doi.org/10.1186/s41747-020-00163-4**

The originally published [1] article contained incomplete funding information. The correct funding information for this publication is as follows:

**Funding**

This study has received funding by European Commission (Horizon 2020) under the grant agreement no. 761031 (nTRACK — H2020-NMBP-2016-2017), the Carlos III Health Institute and the European Regional Development Fund (PI16/02064) and AGAUR from Generalitat de Catalunya (2017SGR1303).
